# Peripheral Neuropathy Instruments for Individuals with Cancer: A COSMIN-Based Systematic Review of Measurement Properties

**DOI:** 10.3390/curroncol31120577

**Published:** 2024-12-06

**Authors:** Silvia Belloni, Arianna Magon, Chiara Giacon, Francesca Savioni, Gianluca Conte, Rosario Caruso, Cristina Arrigoni

**Affiliations:** 1 Department of Public Health, Experimental and Forensic Medicine, Section of Hygiene, University of Pavia, 27100 Pavia, Italy; cristina.arrigoni@unipv.it; 2 Health Professions Research and Development Unit, IRCCS Policlinico San Donato, San Donato Milanese, 20097 Milan, Italy; arianna.magon@grupposandonato.it (A.M.); gianluca.conte@grupposandonato.it (G.C.); 3 Fondazione IRCCS Policlinico San Matteo, 27100 Pavia, Italy; c.giacon@smatteo.pv.it; 4 IRCCS Humanitas Research Hospital, Rozzano, 20089 Milan, Italy; francesca.savioni@humanitas.it; 5 Department of Biomedical Sciences for Health, University of Milan, 20126 Milan, Italy

**Keywords:** peripheral neuropathy, validity, reliability, instruments, patient-reported outcome measure, PROMs, cancer

## Abstract

Although the literature on patient-reported outcomes (PROMs) continues to expand, challenges persist in selecting reliable and valid instruments for assessing peripheral neuropathy (PN) in patients with cancer. This systematic review aimed to identify all validated self-report PN scales and critically appraise their measurement properties. This review was conducted using the COSMIN methodology for PROMs and the PRISMA statement. Five databases were searched from inception to August 2024, identifying 46 eligible studies and 16 PROMs. Evidence quality ranged from “very low” to “moderate”, with notable inconsistencies in the content and structural validity phases of most instruments. Instruments such as the Chemotherapy-induced peripheral neuropathy assessment tool and the Functional Assessment of Cancer Therapy/Gynecologic Oncology Group–Neurotoxicity demonstrated moderate quality and potential utility in clinical practice, while others, including the Location-based assessment of sensory symptoms in cancer and the Measure of Ovarian Symptoms and Treatment, had insufficient evidence to support their use. Importantly, all PROMs focused on chemotherapy-induced peripheral neuropathy, highlighting a significant gap in instruments addressing other PN causes, such as radiotherapy or tumor-related nerve damage. Further research should prioritize developing and validating instruments for distinct cancer populations, ensuring robust psychometric properties and clinical applicability.

## 1. Introduction

Peripheral neuropathy (PN) is a major cancer issue and disabling condition among cancer patients, leading to increasing attention from healthcare systems at a policy level [1,2,3]. This condition affects a great proportion of individuals with cancer, depending on the type of cancer, type of treatment, population, time of diagnosis and prognosis, and genetic and clinical risk factors [4,5,6]. Therefore, the prevalence in the general oncological cancer population is emblematic, especially considering that there are different types of this condition [7], with chemotherapy-induced peripheral neuropathy (CIPN) as the most common [8].

PN is a somatic and autonomic group of signs or symptoms caused by damage to the peripheral nervous system (PNS) or autonomic nervous system (ANS) [9]. PN involves several causes, including metabolic, systemic, and toxic conditions [9]. Most of these causes are related to cancer-related conditions such as nutritional deficiencies (i.e., B1, B6, B12, and vitamin E), damage caused by systemic (i.e., chemotherapy and non-chemotherapy drugs such as immune checkpoint inhibitors and antibody–drug conjugates) or local treatments (i.e., radiotherapy), tumor mass (secondary to compression), paraneoplastic syndromes, trauma injuries (i.e., surgery) and specific malignancies (i.e., multiple myeloma for the secretion of monoclonal protein that directly damages motor and sensorimotor nerve cells by removing their myelin sheaths and causing degeneration of axons) [9].

PN includes a wide range of symptoms, depending on the type and location of the damaged nerves. The most common symptoms and manifestations among patients with cancer are numbness, tingling, and shooting pain or burning, especially in the fingers and toes; others include cramps or muscle wasting, loss of balance with consequent falls [10], dizziness, difficulty walking, clumsiness, difficulty picking up objects or buttoning clothes, facial pain, hearing loss, loss of sensitivity to hot and cold, stomach pain, constipation and diarrhea, sexual alterations, sweating problems, and urination disorders [11,12]. However, depending on the type of nerve affected, neuropathic symptoms and functional disability levels might differ [11].

The spectrum of disorders negatively impacts patients’ quality of life (QoL) [13,14] by interfering with their daily activities [15,16] and sleep [12] even after treatment has concluded [17]. Peripheral neuropathy-related issues might impact patients’ ability to perform daily activities independently, such as opening jars and bottles, standing, walking, and driving, requiring assistance from another person [18,19]. Data from a registry of women with ovarian cancer who received neurotoxic chemotherapy demonstrated that 51% of those were still experiencing CIPN symptoms up to 12 years after the end of treatment [20], and the PN symptom burden remained high even ten years after treatment [21]. Further, PN independently influences the psychosocial dimension, reducing psychosocial adaptation levels [22] and increasing anxiety [23].

Taking into account these data, appropriately recognizing and assessing PN throughout the cancer care pathways is an essential component in oncology care for providing preventive and disease-modifying strategies at early phases to such an extent that familiarity with assessing and reporting PN symptoms represents a fundamental prerequisite of oncologist’ and nurses’ training and curricula [24,25,26]. This aspect becomes particularly relevant given the recent data highlighting high variability and inequalities in oncology symptom management from nurses’ perspective [27]. In this regard, PROs have emerged as the current optimal measure in oncology practice to improve patient communication and symptom monitoring [28,29,30]. Patient-reported outcome measures (PROMs) are standardized questionnaires that collect health outcomes directly from the individuals experiencing them, including symptoms and functional status, without interpreting the patient’s response by a clinician [31].

Although the literature on PROMs continues to broaden, significant challenges remain in selecting reliable and valid tools for specific clinical needs [32,33]. Current systematic reviews (SRs) have primarily focused on evaluating the measurement properties of instruments designed for CIPN alone [34,35,36,37,38,39]. However, these reviews do not address the broader spectrum of PN types experienced by cancer patients. PN in this population can result from various factors beyond chemotherapy, including radiotherapy, tumor-related nerve compression, paraneoplastic syndromes, and surgical trauma, each presenting distinct manifestations. This diversity requires disease-targeted scales that accurately capture these different types of PN. Without a comprehensive understanding and valid assessment of the specific type of neuropathy and patient population, even the most advanced tools may be ineffective because they may fail to detect specific symptoms, mischaracterize the severity or nature of neuropathy, or overlook important variations in how different patient groups experience symptoms [40]. This gap could lead to misinterpretation of patient symptoms, suboptimal clinical decisions, and biased management strategies. Addressing this gap is crucial to ensure patients receive precise evaluations and appropriate care, ultimately improving their quality of life and clinical outcomes. The literature review results focused on consolidating the understanding regarding the quality of the measurement properties of the available tools to assess PN, which could lay the groundwork for targeting the most appropriate instruments for research and practice. Therefore, this systematic review aimed to identify all the available PN scales in addition to those that evaluate CIPN and critically appraise, compare, and summarize the quality of the measurement properties of all self-report peripheral neuropathy questionnaires for adult patients with cancer.

## 2. Materials and Methods

A critical review approach was adopted according to the “COSMIN methodology for systematic reviews of Patient-Reported Outcome Measures” [41] and reported following the PRISMA guidelines [42]. This methodological approach adapts the available Cochrane guidelines regarding the systematic review of interventions, including the GRADE approach for evidence quality evaluation, to the requirements of conducting validation studies. This SR was registered with the International Prospective Register for Systematic Reviews (PROSPERO) with no deviations from the original registered protocol (ID: CRD42024532342).

This SR will address the following questions: (1) What are existing disease-specific peripheral neuropathy-validated instruments for adult patients with cancer? (2) What are the characteristics of the instruments? (3) What is the methodological quality of studies assessing the measurement properties of the instruments? (4) What are the measurement properties and feasibility of the instruments? (5) What are the similarities and differences among the instruments? (6) What are the knowledge and research gaps in this field? Selecting the best PROM for the intended outcome necessitates high-quality studies on the measurement properties of relevant PROMs in the target population and a high-quality SR of measurement property studies in which all information is collected and evaluated systematically and transparently.

The research aim encompasses the four key elements (COSMIN framework) indicated by COSMIN guidelines: the construct (peripheral neuropathy), the population (adults with cancer disease), the type of instrument (all validated self-report peripheral neuropathy scales, including PROMs), and the measurement properties of interest (all measurement properties). To evaluate the methodological quality and measurement properties, we considered the PROM as a whole rather than the single studies focused on a specific PROM, considering that the validation of a questionnaire includes different steps, which may result in multiple publications that complete the entire validation process. The results were qualitatively summarized as no homogeneous sufficient data from different studies on the same measurement property were available. We could not obtain comprehensive knowledge or provide a description of the PROMs’ interpretability and feasibility [41] as several details regarding the related subdomains were lacking.

### 2.1. COSMIN Methodology

The COSMIN initiative provides comprehensive guidelines for conducting systematic reviews of PROMs [41]. COSMIN aims to improve the selection of high-quality PROMs by ensuring that all aspects of their measurement properties are rigorously evaluated. The COSMIN guideline includes a standardized procedure for performing systematic reviews, which is tailored to assess the validity, reliability, and responsiveness of PROMs used in clinical research and practice.

The COSMIN methodology recommends the use of databases like PubMed, EMBASE, Web of Science, Scopus, and CINAHL because these databases provide broad coverage of research in health measurement instruments [41]. PubMed and EMBASE are considered essential due to their extensive indexing of the biomedical literature and emphasis on medical and clinical studies. Additionally, Web of Science and Scopus offer a wider multidisciplinary scope, allowing for the identification of studies that might be missed in more specialized databases. CINAHL is included for its focus on the nursing and allied health literature, making it particularly valuable when evaluating PROMs used in diverse healthcare settings [41]. The selection of these databases is designed to balance comprehensiveness and specificity, enabling the capture of relevant studies across different fields while adhering to the systematic review procedures recommended by COSMIN.

The COSMIN methodology follows a standardized ten-step procedure divided into three main phases [41]. The first phase, the literature search and study selection (Steps 1–4), begins by defining the aim of the review, specifying the construct, population, type of instrument, and measurement properties to be investigated. Next, eligibility criteria are formulated according to the COSMIN framework, ensuring alignment with the review’s objectives. The literature search is then developed and executed using a combination of search terms targeting the construct, population, instrument type, and measurement properties. This phase concludes with the independent screening of abstracts and full-text articles, accompanied by reference checking to ensure all relevant studies are identified.

The second phase, the evaluation of measurement properties (Steps 5–8), focuses on assessing the quality of the selected PROMs [41]. It begins with evaluating the content validity of each PROM, which determines how well the instrument captures the intended construct. The internal structure is then assessed, covering aspects like structural validity, internal consistency, and cross-cultural validity. Following this, other measurement properties are examined, such as reliability, measurement error, criterion validity, construct validity, and responsiveness. Finally, this phase considers the interpretability and feasibility of each PROM, analyzing factors like ease of use, scoring, and relevance to the target population.

The third and final phase, synthesis, recommendations, and reporting (Steps 9–10), involves synthesizing the gathered evidence to formulate recommendations on the most suitable PROMs based on their assessed measurement properties [41]. This phase concludes with preparing the systematic review report, adhering to the COSMIN PRISMA guidelines to ensure transparency and completeness.

The COSMIN methodology also uses specific tools for evaluating the quality of studies and PROMs. The COSMIN Risk of Bias Checklist assesses the methodological quality of studies, rating them on a scale from “very good” to “inadequate” for various measurement properties [41]. The Criteria for Good Measurement Properties are applied to determine whether study findings on each measurement property are sufficient, insufficient, or indeterminate. Additionally, the modified GRADE Approach is used to evaluate the quality of evidence for each measurement property, grading it from “high” to “very low” based on considerations such as risk of bias, inconsistency, imprecision, and indirectness.

Key concepts and terms within the COSMIN framework are central to understanding PROM evaluation. A PROM is an instrument, often a self-administered questionnaire, that allows patients to directly report their health status, providing insight into symptoms, functioning, and overall well-being without clinician interpretation [41]. The quality of a PROM is determined by its measurement properties, which include validity, reliability, and responsiveness. Validity refers to the degree to which a PROM measures what it is intended to measure, encompassing content validity (how well the instrument represents the intended construct), construct validity (the extent to which the PROM correlates with other measures in ways consistent with hypotheses), and criterion validity (how well the PROM aligns with a gold standard measure) [41]. Reliability refers to the consistency of the instrument, including internal consistency (the degree to which items within a scale are related), inter-rater reliability (consistency between different assessors), and test–retest reliability (stability of scores over time). Responsiveness measures a PROM’s sensitivity to detect clinically significant changes over time [41]. While not strictly classified as measurement properties, feasibility and interpretability are also crucial; feasibility relates to the ease of implementing the PROM in various settings, considering factors like time and cost, while interpretability involves the ability to assign meaningful clinical or practical significance to the PROMs’ scores, aiding in their application in research or practice. These aspects collectively guide the assessment of PROMs, ensuring their suitability for capturing patient-reported outcomes in a given context.

### 2.2. Eligibility Criteria

The criteria were formulated in agreement with the four key elements of the review aim. We focused on studies evaluating the features of the PROMs and self-report questionnaires used to evaluate peripheral neuropathy (construct), which were at least partially validated. We included all subjects who underwent any type of cancer treatment (i.e., new treatment agents and radiotherapy), including peripheral neuropathy caused by tumor growth and pressure on nerves and peripheral neuropathy as a consequence of cancer surgery (given by nerve damage). This approach considers the concept of cancer-related peripheral neuropathy that can be caused by cancer disease or its treatments. This criterion allowed us to investigate a wide range of measurements that can be more appropriate for specific populations, considering that there are clearly distinct mechanisms of action at the base and different manifestations that can be detected by scales differently.

Studies were included if they: (a) reported a disease-specific peripheral neuropathy instrument(s) designated for adult patients with cancer, including hematological malignancies, (b) described the processes of the development and validation of an instrument(s), (c) had full-text availability, and (d) were scientific methodological peer-reviewed papers. Accordingly, the grey literature was not considered in this review as the aim was to find any relevant peer-reviewed scale to be employed in future clinical trials. Studies were excluded if they: (a) were not validation studies (i.e., case studies, reports, discussion papers, letters, editorials, SRs, study protocols, and published conference abstracts), (b) were trials that used the instruments only for outcome measurement, (c) were studies using animals as a population or preclinical studies, (d) were focused on the pediatric population as it has a distinct clinical phenotype and long-term course, and (e) were scientific peer-reviewed methodological studies.

### 2.3. Search Strategy

The initial search was conducted in April 2024, covering studies from database inception to that date, to ensure a comprehensive retrieval of relevant research. The search included five databases, PubMed, EMBASE, Web of Science, Scopus, and CINAHL, selected according to the COSMIN recommendations (Supplementary Table S1) [43,44,45]. We developed our search strategy following the COSMIN recommendations to ensure a comprehensive and sensitive retrieval of relevant studies. Our approach involved a combination of broad and focused search terms tailored to each database’s unique indexing and structure. Specifically, we utilized search filters that targeted studies reporting on the development, validation, and psychometric properties of PROMs for PN in cancer patients. For databases like PubMed, we used a sensitive search approach, which included MeSH terms and free-text words across all fields to maximize the retrieval of relevant studies. For other databases, such as EMBASE, Web of Science, Scopus, and CINAHL, we adapted our search strings to ensure compatibility with their respective search functionalities, employing title–abstract–keyword searches when necessary [46,47].

We applied the “all fields” filter in cases where a broader search scope was needed, ensuring that all relevant mentions of the terms were considered, regardless of the field in which they appeared. The exclusion criteria resulted in the removal of studies focused on pediatric populations and caregivers, which were applied uniformly across databases to maintain consistency with this review’s eligibility criteria. Additionally, we adapted phrases with more than one word in constructing search queries based on each database’s requirements. Additionally, in constructing search strings, we adapted phrases with more than one word based on each database’s requirements; in some cases, inverted commas were not used if the database treated these phrases as default search strings. This methodical approach, as reflected in the detailed queries shown in Supplementary Table S1, ensured a balance between sensitivity and specificity, allowing for a thorough identification of relevant studies while minimizing the retrieval of non-relevant results. 

To maintain the currency of this review, we conducted a final update of the literature search before this review’s submission in August 2024. This update ensured that no recent publications were missed. The entire search process was not limited by language, and when non-English records were identified, web-based tools were used to convert HTML text into English for initial screening and assessment. This approach ensured that relevant studies in various languages were considered, allowing for a more comprehensive review of the available literature. To supplement our database search, we used Google Scholar to identify any additional relevant publications. We ensured that studies selected through Google Scholar were peer-reviewed by verifying the publication source, and only those from peer-reviewed journals were considered. Additionally, any studies identified through Google Scholar were cross-checked with those from other databases to confirm their relevance and adherence to our inclusion criteria.

### 2.4. Screening and Data Extraction (Selection and Coding)

We identified and removed the duplicates retrieved from the databases using Zotero software (version 6.0.36). Then, the abstract and full-text screening was conducted blindly in Rayyan’s review software by two authors who selected the full texts separately for final inclusion. If a study was relevant based on the title and abstract screening for at least one reviewer, we retrieved the full text for the assessment. The two researchers’ disagreements were resolved through conversation, and a consultation with a third author was adopted as necessary. To include all potentially relevant studies, we identified for full-text screening all validation studies using PROMs on the population of interest in case the outcome assessed was not clearly defined in the abstract.

Two authors performed the extraction separately [48], and uncertain cases were discussed. Before extracting the data, we conducted a pilot extraction form review to ensure inter-rater agreement. The authors extracted data on the characteristics of the PROM(s), the characteristics of the included sample(s), the results on measurement properties, and the information about the interpretability and feasibility of the PROM score(s).

To obtain a full description of each PROM’s applicability and feasibility, we extracted information regarding the (a) PROM identification and authors, (b) country of origin, (c) population (i.e., cancer diagnoses) and sample size, (d) treatment exposure, (e) construct, (f) nerve damage and impairment, (g) investigated domains, (h) longitudinal validity, (i) number of items, (j) intent and purpose of the scale, (k) available translations/versions, and (l) measurement properties of the included instruments (i.e., content validity, structural validity, internal consistency, cross-cultural validity, measurement invariance, reliability, measurement error, criterion validity, construct validity, responsiveness).

### 2.5. Methodological Quality Assessment of Instruments (Risk of Bias)

Two independent reviewers used the validated COSMIN checklist to evaluate the methodological quality of the included studies [49,50,51]. The risk of bias checklist includes the evaluation of ten domains: PROM development, content validity (i.e., an evaluation of questionnaire comprehensibility), structural validity (it refers to the internal structure, including the number of domains and items within the instrument), internal consistency (an indicator of whether or not the items in a survey measure what they are intended to measure), cross-cultural validity/measurement invariance (it examines whether metrics developed in a single culture are applicable), reliability (is the extent to which an instrument would provide the same results if the measurement were carried out repeated under the same conditions), measurement error (the difference between the true value of a variable and the value obtained by a questionnaire), criterion validity (how well a test correlates with an established standard of comparison called criterion), hypotheses testing for construct validity (if the research findings are consistent with the theoretically established objectives), and responsiveness (the ability of a questionnaire to detect clinically significant changes over time) [52].

Each measurement property was evaluated with 3–35 items, and each item was rated on a five-level scale: “very good”, “adequate”, “doubtful”, “inadequate”, and “not applicable”. Each study’s overall quality rating is based on the lowest rating of any standard in the box, using the “worst score counts” approach [50]. For example, if any item in a box is scored poorly, the methodological quality score for the box domain will be poor [53]. However, the COSMIN framework prioritizes content validity as the primary feature of instrument validation [54]. Any disagreement between the two authors was resolved by a third to obtain a definitive conclusion.

### 2.6. Quality of the PROMs’ Measurement Properties and Synthesis

The results of each study were evaluated independently against the criteria of good measurement properties as sufficient (+), insufficient (–), or indeterminate (?). The measurement properties included structural validity, internal consistency, reliability, measurement error, hypotheses testing for construct validity, cross-cultural validity/measurement invariance, criterion validity, and responsiveness. These results were then included in the table of the PROMs’ measurement properties. According to the COSMIN manual recommendations [49,51], we rated the domains by basing the PROMs’ judgment on the previously published COSMIN SR [38].

### 2.7. Quality of Evidence and Recommendations

The modified GRADE version was applied to assess the overall evidence quality of each PROM [41]. While the GRADE approach was designed to assess the evidence quality of intervention studies by including five domains (risk of bias, indirectness, inconsistency, imprecision, and publication bias), the modified GRADE approach for grading the quality of the evidence in systematic reviews of PROMs comprised four domains in relation to validation study phases. This methodology consists of grading evidence quality as “high”, “moderate”, “low”, or “very low” [41,49]. Four domains were considered in determining evidence quality: (1) risk of bias, (2) inconsistency, (3) imprecision, and (4) indirectness. A “high” level of evidence is when “we are very confident that the true measurement property lies close to that of the estimate of the measurement property”, “moderate” when “we are moderately confident in the measurement property estimate: the true measurement property is likely to be close to the estimate of the measurement property, but there is a possibility that it is substantially different”, “low” when “our confidence in the measurement property estimate is limited: the true measurement property may be substantially different from the estimate of the measurement property”, and “very low” when “we have very little confidence in the measurement property estimate: the true measurement property is likely to be substantially different from the estimate of the measurement property”.

Recommendations on the most appropriate PROM for evaluative application were formulated with regard to the construct of interest and study population. According to the COSMIN guideline [41], the included instruments were categorized into three distinct categories based on their measurement properties: (A) instruments with evidence for sufficient content validity (any level) and at least low-quality evidence for sufficient internal consistency, (B) instruments categorized not in A or C, and (C) instruments with high-quality evidence for an insufficient measurement property [41].

### 2.8. Measures to Control Bias in the Review Process

To ensure the rigor and reliability of this systematic review, we implemented several measures to minimize potential sources of bias throughout the review process.

Firstly, the screening of abstracts and full-text articles was conducted independently by two reviewers to ensure objectivity in the selection of studies (SB and FS). This dual-review approach helped reduce selection bias by ensuring that study inclusion was based on a consensus of independent evaluations. Any disagreements during the screening process were resolved through discussion, and a third reviewer was involved when consensus could not be reached (RC). This approach aimed to ensure that all studies considered for inclusion were evaluated impartially.

Secondly, we used the COSMIN Risk of Bias checklist as part of our quality assessment of the included studies. This standardized tool provided a systematic way to evaluate the methodological quality and potential biases in the measurement properties of PROMs. Using this checklist ensured consistency and objectivity in assessing study quality across all included studies. Additionally, we established clear, predefined eligibility criteria for study inclusion and exclusion. These criteria, developed before conducting the literature search, were applied uniformly across all databases, helping to minimize selection bias and ensure that studies were included based solely on their relevance to this review’s objectives.

To further mitigate the risk of bias, we conducted a pilot test of the data extraction form before full data extraction began. This pilot process ensured that the reviewers had a consistent understanding of the data elements to be extracted and helped to reduce the risk of extraction errors or inconsistencies.

Moreover, we did not impose language restrictions during the selection process, allowing for the inclusion of relevant studies published in any language. This measure helped reduce language bias, although we acknowledge that most studies were identified through English-language search terms due to the databases’ indexing. Finally, we conducted additional searches using Google Scholar and performed reference list checks to identify any potentially relevant studies that might have been missed during the initial database searches. This supplementary searching process served as a safeguard to ensure that all relevant evidence was considered, thereby reducing the risk of publication bias and enhancing the comprehensiveness of this review.

## 3. Results

A total of 1515 results were identified from the database search. After removing duplicates, 1473 records were exported to Rayyan software (© 2022 RAYYAN) for screening. The selection process led to the identification of 46 eligible studies and 16 PROMs (not including the different versions). Supplementary Figure S1 depicts the sources, the entire selection process, and the reasons for excluding records. The PROMs included all validated self-report scales used to assess peripheral neuropathy in adults with cancer.

### 3.1. Characteristics of the Included Studies

The characteristics of the included studies are reported in Supplementary Table S2. This table lists the included PROMs along with the corresponding references. The choice of presenting the table based on PROMs rather than single studies was based on the amount of literature, which would have rendered the presentation redundant. All the scales had an evaluative intent with a clear description of the context to assess peripheral neuropathy in patients undergoing neurotoxic chemotherapy.

The construct of interest was CIPN for the majority of the PROMs, except for the Korean version of the Neurotoxicity 4-item (K-NTX-4) scale [55], Location-based assessment of sensory symptoms in cancer (L-BASIC) [56], and Measure of Ovarian Symptoms and Treatment-26 items (MOST-S26) [57] that also assessed disease-related symptoms. Only one PROM validation (the PRO-CTCAE-CIPN) [58,59,60,61,62] involved patients undergoing surgery and radiotherapy in addition to chemotherapy. However, in all the samples, the majority of patients received neurotoxic chemotherapy. Overall, the studies focused on adults with solid and hematologic tumors. Four PROMs, the Chemotherapy-induced peripheral neuropathy assessment tool (CIPNAT) [63,64,65,66,67], the European Organization of Research and Treatment of Cancer-Quality of Life Questionnaire-Twenty-item scale (EORTC-QLQ-CIPN15/20 fifteen/twenty-item scale) [68,69,70,71,72,73,74,75,76,77,78,79], the Functional Assessment of Cancer Therapy/Gynecologic Oncology Group–Neurotoxicity (FACT-GOG-Ntx) [80,81,82,83,84,85,86,87], and the Peripheral Neuropathy Questionnaire (PNQ) [88,89], underwent to a cross-cultural validation and therefore are available in multiple languages. Functional impairment involves sensory–motor and autonomic nerves, which impact the physical, functional, emotional, and social domains. Two PROMs (the FACT-GOG-Ntx and the EORTC-QLQ-CIPN15/CIPN20 (fifteen/twenty-items scale) were tested within the context of randomized clinical trials). The Treatment-Induced Neuropathy Assessment Scale (TNAS v.1, v.2,v.3) [90,91], the PRO-CTCAE-CIPN [58,59,60,61,62], the PNQ [88,89], the Oxaliplatin-Associated Neurotoxicity Questionnaire (OANQ) [92,93], MOST-S26 [57], the L-BASIC [56], the K-NTX-4 [55], the Indication for CTC Grading of Peripheral Neuropathy Questionnaire (ICPNQ) [94], the FACT-GOG-Ntx [80,81,82,83,84,85,86,87], the EORTC-QLQ-CIPN20/CIPN15 [68,69,70,71,72,73,74,75,76,77,78,79], the Chemotherapy-induced peripheral neuropathy-Rasch-built Overall Disability Scale (CIPN-R-ODS) [95], and the Chemotherapy-induced peripheral neuropathy integrated assessment—oxaliplatin subscale (CIPNIA-OS) [96] applied a longitudinal design, which enabled us to investigate the longitudinal validity.

### 3.2. The Methodological Quality of Instruments (Risk of Bias)

The methodological quality of the included PROMs was assessed using the COSMIN Risk of Bias checklist and according to the COSMIN manual recommendations. Discrepancies between the two researchers arose in specific items when some details were missing in the studies; this deficiency implied that one researcher rated the item as “doubtful” and the other as “inadequate”. However, the two researchers’ final judgment of the domains was congruent. The table structure of the results reflects the corresponding COSMIN boxes’ methodological structure of the manual [49]. A detailed overview of the methodological quality of each study is reported in Table 1, which was created according to the COSMIN manual suggestions.

Most of the studies had deficiencies in the content and structural validity phases, as few followed the recommended criteria for item development and content validation. Only seven PROMs included cognitive interviews for item development, and only five [62,68,80,90,97] employed an appropriate qualitative data collection method to find relevant items. Only five PROMs [68,69,90,97,98] asked patients and professionals about the content validity domain’s relevance, comprehensiveness, and comprehensibility. However, none of the PROMs included a complete content validation step. The responsiveness domain was judged as “not applicable” as no gold standard exists. However, most scales compared the investigated scale with other outcome measurement instruments. None of the questionnaires rigorously reported all the phases necessary for developing and validating a questionnaire [62,68,80,90,97].

### 3.3. Quality of the PROMs’ Measurement Properties and Synthesis

The quality of the PROMs’ measurement properties and synthesis are presented in Table 2 and Table 3. For the content validity subdomains, we rated them as indeterminate (?) when the authors did not conduct the steps for testing the content validity of the questionnaire, such as asking patients or professionals (target population) about the relevance and comprehensibility of the items. However, in these cases, as the content validity domain is the most relevant aspect in scale development and validation research, we judged the overall content validity rating domain as insufficient (−). This position enabled us to align with the overall judgment from previous research on PROMs [38]. None of the PROMs addressed measurement errors. Criterion validity with the gold standard was not tested as there is no established gold standard for CIPN testing [73].

### 3.4. Overall Quality of Evidence and Recommendations

Two researchers rated the overall quality of evidence for each PROM based on the methodological quality and the measurement property rating. No discrepancies were encountered in the rating process between the two researchers. Seven PROMs (L-BASIC, PNQ, CINQ, OANQ, K-NTX-4, MOST-S26, CIPNIA-OS, and CIPN Self-check) were graded as “very low”, four PROMs (TNAS v.1–v.3, ICIPNQ, PRO-CTCAE, and CAS-CIPN) as “low”, and four (CIPN-R-ODS, EORTC-QLQ-CIPN20/CIPN15, FACT-GOG-Ntx, and CIPNAT) as “moderate”.

Based on the quality of the evidence, the L-BASIC, PNQ, CNQ, AONQ, K-NTX-4, MOST-26, CIPNIA-OS, and CIPN Self-check [99] were recommended as C (not be recommended for use), whereas the remaining instruments (CIPN-R-ODS, EORTC-QLQ-CIPN20/CIPN15, TNAS v.1–v.3, ICPNQ, FACT-GOG-Ntx, CIPNAT, PRO-CTCAE, and CAS-CIPN) were recommended for use but required further investigations. The main reason for this judgment was based on the fact that none of the instruments applied the required steps for testing the content and structural validity (Table 4).

## 4. Discussion

This study examined 46 eligible articles and found 16 PROMs used to assess peripheral neuropathy in adult patients with cancer. We found three additional instruments (L-BASIC, MOST-26, CIPNIA-OS) compared to a recent SR assessing chemotherapy-induced peripheral neuropathy with patient-reported outcome measures [38].

Appropriately assessing PN throughout the cancer care pathways is an essential component of oncology care, considering that this symptom frequently results in treatment dose reduction, cessation, and prolonged infusion times [4,6], along with effects on functional impairment and QoL [14]. PROs have been recognized as the most effective measure in oncology practice to improve symptom management and survival of patients with cancer [100]. Although several SRs have been conducted to evaluate the measurement properties of CIPN scales, our aim was to identify all the available PN scales in addition to those that evaluate CIPN and critically appraise the quality of the measurement properties of all self-report questionnaires for adult patients with cancer. This aspect is particularly important in oncology care, where the same disorder may have several dimensions [101], causes, and underlying mechanisms, resulting in various presentations called “phenotypic characterization” in symptom science research [102].

We found that all the validated questionnaires focused on CIPN, and no validated questionnaires exist evaluating peripheral neuropathy caused by other conditions, such as radiotherapy, tumor mass compression, paraneoplastic syndromes, trauma injuries from surgery, and specific malignancies (i.e., multiple myeloma). These situations may lead to different presentations of peripheral neuropathy [10,12]. Although several studies examined disease-related symptoms and patients undergoing surgery and radiotherapy, the underlying construct of interest was CIPN. When developing a scale, items should be designed based on individuals’ experiences and assessing existing indicators of the domain [103]. Therefore, a scale should reflect the specific condition to ensure the quality of construct measurement and be able to detect and assess the specific presentation [103].

Assessing the methodological quality of an instrument is crucial as it impacts the reliability of the results [49]. Our SR found that none of the instruments measuring peripheral neuropathy satisfied all the COSMIN development quality criteria [49]. The main inadequacy was found in the item development and the content validation phases. Content validity is considered the most relevant measurement property as it regards the clarity and comprehensiveness of items with respect to the construct of interest and target population [50]. In our analysis, all the studies used a literature review as a deductive method for item generation, whereas qualitative inductive methods, such as cognitive interviews, focus groups, and consensus discussions with experts, were not used in the majority of PROMs. Best practice suggests combining deductive and inductive methods to define the domains and questions [103]. According to the COSMIN guidelines, each instrument should be evaluated based on rigorous criteria reflecting each validation study’s methodological quality [41]. The development and validation of a questionnaire requires investigators’ thorough consideration of steps that guarantee the reliability and validity of the instrument [103,104,105]. This evaluation enables clinicians and researchers to detect the most appropriate and reliable tool to be employed within the relevant clinical contexts to assess the conditions [103].

Regarding the overall quality of evidence, the L-BASIC, PNQ, CINQ, OANQ, K-NTX-4, MOST-S26, CIPNIA-OS, and CIPN Self-check were graded as “very low”, the TNAS v.1–v.3, ICIPNQ, PRO-CTCAE, and CAS-CIPN as “low”, and the CIPN-R-ODS, EORTC-QLQ-CIPN20/CIPN15, FACT-GOG-Ntx, and CIPNAT as “moderate”. Although some of these instruments may be considered valuable, none were of high-quality evidence for inconsistencies in the content and structural validity phases. This hampers us from recommending a valid and reliable tool currently employed in clinical practice. In summary, the CIPNAT scale seems to be the most valid and reliable tool, considering the results of the validation steps and measurement properties. However, the number of items (50) undermines the feasibility of the instruments in clinical practice. Considering a recent and similar SR [38], our results seem to underestimate the quality evidence of the included PROMs. Although we acknowledge the accuracy of these results, we chose to be conservative and suggest caution in the interpretability of the findings in light of the previous considerations on the PROMs’ methodological quality and measurement properties results.

This study has certain limitations that should be taken into consideration. Firstly, we chose not to provide a single qualitative scale description, which limits our comprehensive understanding of each PROM and interpretation of the methodological outcomes in light of the instruments’ characteristics. However, we performed a COSMIN rather than a general systematic review, which required specific attention to the methodological quality and measurement properties. Secondly, we could not discuss the interpretability and feasibility of the PROMs, as additional details were essential to comprehensively understand these properties. Thirdly, while we identified several additional studies through other methods, it is possible that our search strategy was not sensitive enough to detect all relevant papers. Nevertheless, the final results encompassed additional PROMs compared to a recently published COSMIN review [38], and that review provided valuable context that helped us supplement our findings. Fourthly, only two authors were involved in evaluating measurement properties; however, a third author was consulted in cases of disagreement. Finally, although we adhered to a rigorous methodological approach, the evaluation could yield different results if conducted by other researchers. Fifthly, while our search strategy utilized English-language terms due to the indexing and search functionalities of the selected databases, we did not impose a language restriction during the selection process. This means that non-English studies with English abstracts were still identified and considered for inclusion. However, this approach may have limited the sensitivity of the search in capturing non-English studies that did not provide an English abstract. To mitigate this, we used translation tools when necessary to assess non-English full-text articles. Another limitation is that some relevant studies were identified through supplementary methods, such as Google Scholar, rather than the primary database searches. This gap between the studies found in Google Scholar and those identified through our database queries may reflect that our search strategy was designed to be more specific than sensitive to improve the feasibility and manageability of the search process. While this specificity helped to focus on highly relevant studies, it may have led to some relevant records being initially missed. However, we mitigated this limitation by conducting an additional search in Google Scholar and performing reference checks, ensuring that these studies were not overlooked in the final review.

## 5. Conclusions

This systematic review highlights that although a substantial body of literature focuses on the development of PROMs for CIPN, none of the instruments examined fully adhered to the necessary steps of the PROM validation process, particularly in the critical phases of content and structural validity. Instruments like the CIPNAT and FACT-GOG-Ntx exhibit moderate methodological quality and measurement properties, but none achieve the high standards necessary for confident clinical application. These findings underscore an urgent need for rigorous, large-scale studies to enhance the validity and reliability of the existing tools. Moreover, subgroup characteristics such as multimorbidity (e.g., co-existing conditions like Raynaud’s syndrome), prior cancer diagnoses, and varying cancer treatments (e.g., immunotherapies or targeted therapies) play a pivotal role in shaping the presentation and progression of peripheral neuropathy. These factors significantly influence symptom patterns, severity, and patient experiences, underscoring the importance of tailoring PROMs to capture these variations effectively.

Future PROM development must address these diverse etiologies and patient-specific profiles to ensure instruments accurately reflect the complexity of neuropathy in oncology. To achieve this, PROMs should integrate insights from diverse patient populations, accounting for variations in cancer type, treatment history, gender, age, and comorbid conditions. Comparative psychometric studies are essential to evaluate and benchmark instrument performance, guiding clinicians toward the most suitable tools for precise symptom assessment and effective intervention. A broader, more nuanced spectrum of PROMs is crucial to improving symptom management and reducing the burden of PN among cancer patients.

## Figures and Tables

**Table 1:** Methodological quality of instruments.

PROM										
	Content Validity		Internal Structure		Remaining Measurement Properties					
	PROM Development	Content Validity	Structural Validity	Internal Consistency	Cross-Cultural Validity	Reliability	Measurement Error	Criterion Validity	Construct Validity	Responsiveness
L-BASIC	I	I	I	V	N	A	I	I	A	N
PNQ	I	I	I	I	A	I	I	V	A	N
CIPN-R-ODS	I	I	A	V	N	A	I	I	I	N
EORTC-QLQ-CIPN20/CIPN15	A	A	A	V	A	A	I	V	A	N
TNAS v.1, v.2 and	A	A	I	V	N	I	I	V	I	N
TNAS v.3	A	A	I	A	N	A	I	A	A	N
CINQ	I	I	I	A	N	A	I	I	A	N
OANQ	I	I	I	A	N	A	I	I	A	N
ICPNQ	I	I	I	V	N	A	I	I	A	N
FACT-GOG-Ntx	A	A	A	V	A	I	I	V	A	N
K-NTX-4	I	I	I	A	I	A	I	A	I	N
CIPNAT	A	A	A	V	A	A	I	A	A	N
MOST-S26	I	I	I	I	N	I	I	V	A	N
PRO-CTCAE	A	I	I	V	N	A	I	A	A	N
CIPNIA-OS	I	I	A	V	N	A	I	V	I	N
CAS-CIPN	A	A	A	V	N	I	I	V	A	N
CIPN Self-check	I	I	I	I	N	I	I	I	I	N

Legend: Comprehensive Assessment Scale for Chemotherapy-induced Peripheral Neuropathy (CAS-CIPN); Chemotherapy-Induced Neurotoxicity Questionnaire (CINQ); Chemotherapy-induced peripheral neuropathy self-check sheet (CIPN self-check sheet); Chemotherapy-induced peripheral neuropathy assessment tool (CIPNAT); Chemotherapy-induced peripheral neuropathy integrated assessment—oxaliplatin subscale (CIPNIA-OS); Chemotherapy-induced peripheral neuropathy-Rasch-built Overall Disability Scale (CIPN-R-ODS); European Organization of Research and Treatment of Cancer-Quality of Life Questionnaire-Twenty-item scale (EORTC-QLQ-CIPN20/CIPN15 fifteen/twenty-item scale); Functional Assessment of Cancer Therapy/Gynecologic Oncology Group–Neurotoxicity (FACT-GOG-Ntx); Indication for CTC Grading of Peripheral Neuropathy Questionnaire (ICPNQ); Korean version of the Neurotoxicity 4-item (K-NTX-4); Location-based assessment of sensory symptoms in cancer (L-BASIC); Measure of Ovarian Symptoms and Treatment-26 items (MOST-S26); Oxaliplatin-Associated Neurotoxicity Questionnaire (OANQ); Patient Neurotoxicity Questionnaire (PNQ); Patient-reported Outcome-Common Terminology Criteria for Adverse Events (PRO-CTCAE); Treatment-Induced Neuropathy Assessment Scale (TNAS); inadequate (I); very good (V); not applicable (N); adequate (A).

**Table 2:** Measurement properties assessment of the instruments.

PROM									
	Content Validity Rating	Structural Validity	Internal Consistency	Cross-Cultural Validity/Measurement Invariance	Reliability	Measurement Error	Criterion Validity **	Construct Validity	Responsiveness
CAS-CIPN	-	+	+	?	?	?	?	+	?
CINQ	-	?	+	?	+	?	?	?	?
CIPN Self-check	-	?	?	?	?	?	?	+	?
CIPNAT	+	-	+	+/-	+	?	?	+	?
CIPNIA-OS	-	+	+	?	+	?	?	?	?
CIPN-R-ODS	+	+	+	?	?	?	?	?	?
EORTC-QLQ-CIPN20/CIPN15	+	-	+	+	+	?	?	+	+
FACT-GOG-Ntx	+	-	+	+/?	?	?	?	+	-
ICPNQ	-	?	+	?	+	?	?	+	?
K-NTX-4	-	?	+	?	+	?	?	-	?
L-BASIC	-	-	+	?	+	?	?	+	-
MOST-S26	-	?	?	?	?	?	?	+	+
OANQ	-	-	+	?	+	?	?	?	?
PNQ	-	-	-	+/-	?	?	?	+	+
PRO-CTCAE	+	?	?	?	+/-*	?	?	+	+
TNAS v.1, v.2	+	?	+	?	?	?	?	?	-
TNAS v.3	+	?	+	?	+	?	?	-	?

Legend: “+” = sufficient, “-” = insufficient, “?” = indeterminate; * + for severity of symptoms and - for intensity of symptoms; ** No agreed gold standard in CIPN testing exists; Comprehensive Assessment Scale for Chemotherapy-induced Peripheral Neuropathy (CAS-CIPN); Chemotherapy-Induced Neurotoxicity Questionnaire (CINQ); Chemotherapy-induced peripheral neuropathy self-check sheet (CIPN self-check sheet); Chemotherapy-induced peripheral neuropathy assessment tool (CIPNAT); Chemotherapy-induced peripheral neuropathy integrated assessment—oxaliplatin subscale (CIPNIA-OS); Chemotherapy-induced peripheral neuropathy-Rasch-built Overall Disability Scale (CIPN-R-ODS); European Organization of Research and Treatment of Cancer-Quality of Life Questionnaire-Twenty-item scale (EORTC-QLQ-CIPN15/CIPN20 fifteen/twenty-item scale); Functional Assessment of Cancer Therapy/Gynecologic Oncology Group–Neurotoxicity (FACT-GOG-Ntx); Indication for CTC Grading of Peripheral Neuropathy Questionnaire (ICPNQ); Korean version of the Neurotoxicity 4-item (K-NTX-4); Location-based assessment of sensory symptoms in cancer (L-BASIC); Measure of Ovarian Symptoms and Treatment-26 items (MOST-S26); Oxaliplatin-Associated Neurotoxicity Questionnaire (OANQ); Patient Neurotoxicity Questionnaire (PNQ); Patient-reported Outcome-Common Terminology Criteria for Adverse Events (PRO-CTCAE); Treatment-Induced Neuropathy Assessment Scale (TNAS).

**Table 3:** Results of the PROM’s measurement properties.

PROM									
	Sample Size	Structural Validity	Internal Consistency	Cross-Cultural Validity/Measurement Invariance	Reliability	Measurement Error	Criterion Validity	Construct Validity *	Responsiveness *
CAS-CIPN	327	CFA supported a one-factor structure (RMSEA = 0.079), indicating unidimensionality.	Cronbach α = 0.826	N.I.	N.I.	N.I.	N.A.	A total of 1/1 hypotheses supported. Higher scores are associated with increased neuropathy severity.	N.I.
CINQ	23	N.I.	Cronbach α = 0.84–0.94	N.I.	ICC = 0.1–1.0	N.I.	N.A.	N.I.	N.I.
CIPN Self-check sheet	248	N.I.	N.I.	N.I.	N.I.	N.I.	N.A.	A total of 1/1 hypothesis supported. The self-check sheet scores are correlated with clinical assessments of neuropathy severity.	N.I.
CIPNAT	735	CFA with CFI = 0.98; RMSEA = 0.07 (Turkish version), indicating good model fit.	Cronbach α = 0.90 (original version)	No important differences observed across different cultural versions.	Correlation between tests = 0.89–0.93, indicating high test–retest reliability	N.I.	N.A.	A total of 3/3 hypotheses supported, indicating that the tool accurately differentiates between groups with varying levels of neuropathy.	N.I.
CIPNIA-OS	186	EFA supported a seven-factor structure; the final model retained these seven domains to capture different aspects of neuropathy symptoms.	Cronbach’s α = 0.764, indicating acceptable internal consistency	N.I.	ICC = 0.99, indicating very high test–retest reliability	N.I.	N.A.	N.I.	N.I.
CIPN-R-ODS	281	Rasch analysis performed, with χ^2^ = 0.58, indicating good fit to the Rasch model.	Pearson separation index = 0.92, suggesting high internal consistency	N.I.	N.I.	N.I.	N.A.	N.I.	N.I.
EORTC-QLQ-CIPN20/CIPN15	2208	CFA did not support the initial three-factor structure; model adjustments were required to improve fit.	Cronbach’s α = 0.73–0.91 across subscales, indicating good to excellent internal consistency	N.I.	Correlation between tests = 0.73–0.86, indicating moderate to high test–retest reliability	N.I.	N.A.	A total of 14/15 hypotheses supported, indicating that the measure accurately differentiates between groups with varying neuropathy severity (e.g., patients with different levels of symptoms). Cohen’s d = 0.52, representing a moderate effect size.	A total of 3/3 hypotheses supported for detecting changes over time, suggesting that the instrument is responsive to changes in patient-reported neuropathy symptoms.
FACT-GOG-Ntx	>994	CFA did not support the proposed four-factor structure, indicating issues with model fit and the need for further refinement.	Cronbach’s α = 0.82–0.91, indicating good to excellent internal consistency across subscales	No important differences observed across cultural adaptations.	N.I.	N.I.	N.A.	A total of 7/8 hypotheses supported, indicating that the tool can differentiate between treated and untreated groups regarding neuropathy symptoms.	A total of 6/7 hypotheses supported, suggesting that the instrument is sensitive to detecting changes in neuropathy symptoms over time.
ICPNQ	156	N.I.	Cronbach’s α = 0.84 (sensory), 0.74 (motoric), and 0.61 (autonomic), indicating good internal consistency for sensory and motoric scales but lower reliability for the autonomic scale	N.I.	ICC = 0.83, suggesting good test–retest reliability	N.I.	N.A.	A total of 2/2 hypotheses supported, indicating that the questionnaire accurately differentiates between patients with varying levels of neuropathy severity.	N.I.
KNT-X-4	237	N.I.	Cronbach’s α = 0.80, indicating good internal consistency	N.I.	ICC = 0.84, suggesting good test–retest reliability	N.I.	N.A.	A total of 0/1 hypothesis supported, indicating that the measure does not fully meet expectations for differentiating between groups.	N.I.
L-BASIC	97	N.I.	Cronbach’s α = 0.74, indicating acceptable internal consistency	N.I.	Kappa (K) = 0.76–0.88, indicating substantial agreement and good test–retest reliability	N.I.	N.A.	A total of 2/2 hypotheses supported, indicating that the tool successfully differentiates between groups with varying levels of neuropathy.	N.I.
MOST-S26	726	N.I.	N.I.	N.I.	N.I.	N.I.	N.A.	A total of 6/7 hypotheses supported, indicating that the instrument can accurately differentiate between patient groups with varying levels of symptoms.	A total of 2/2 hypotheses supported, suggesting that the instrument is capable of detecting changes in symptoms over time.
OANQ	23	N.I.	Cronbach’s α = 0.90, indicating excellent internal consistency	N.I.	ICC = 0.1–1.0, indicating a wide range in test–retest reliability	N.I.	N.A.	N.I.	N.I.
PNQ	100		Cronbach’s α = 0.69 (mean of time points), indicating acceptable internal consistency	No important differences				A total of 1/1 hypothesis supported. Higher scores are correlated with clinical measures of neuropathy severity.	AUC = 1 (sensory part) and AUC = 0.9 (motor part), indicating high ability to distinguish between patients with varying degrees of sensory and motor neuropathy
PRO-CTCAE-CIPN	975	N.I.	N.I.	N.I.	ICC = 0.55 (interference) and 0.80 (severity), indicating good reliability for severity assessments but moderate reliability for interference	N.I.	N.A.	A total of 4/4 hypotheses supported, indicating that the tool can differentiate between groups with varying levels of chemotherapy-induced peripheral neuropathy symptoms.	N.I.
TNAS v.1–v.2	186	Cluster analysis performed to identify item groupings related to neuropathy symptoms.	Cronbach’s α = 0.80–0.87, indicating good internal consistency	N.I.	N.I.	N.I.	N.A.	N.I.	A total of 1/2 hypotheses supported, indicating that the instrument has partial ability to detect changes in neuropathy symptoms over time.
TNAS v.3	163		Cronbach’s α = 0.88–0.90, indicating excellent internal consistency	N.I.	ICC = 0.97, indicating very high test–retest reliability	N.I.	N.A.	A total of 1/2 hypotheses supported, indicating that the measure partially meets expectations for differentiating between groups.	N.I.

Legend: Comprehensive Assessment Scale for Chemotherapy-induced Peripheral Neuropathy (CAS-CIPN); Chemotherapy-Induced Neurotoxicity Questionnaire (CINQ); Chemotherapy-induced peripheral neuropathy self-check sheet (CIPN self-check sheet); Chemotherapy-induced peripheral neuropathy assessment tool (CIPNAT); Chemotherapy-induced peripheral neuropathy integrated assessment—oxaliplatin subscale (CIPNIA-OS); Chemotherapy-induced peripheral neuropathy-Rasch-built Overall Disability Scale (CIPN-R-ODS); European Organization of Research and Treatment of Cancer-Quality of Life Questionnaire-Twenty-item scale (EORTC-QLQ-CIPN15/CIPN20 fifteen/twenty-item scale); Functional Assessment of Cancer Therapy/Gynecologic Oncology Group–Neurotoxicity (FACT-GOG-Ntx); Indication for CTC Grading of Peripheral Neuropathy Questionnaire (ICPNQ); Korean version of the Neurotoxicity 4-item (K-NTX-4); Location-based assessment of sensory symptoms in cancer (L-BASIC); Measure of Ovarian Symptoms and Treatment-26 items (MOST-S26); Oxaliplatin-Associated Neurotoxicity Questionnaire (OANQ); Patient Neurotoxicity Questionnaire (PNQ); Patient-reported Outcome-Common Terminology Criteria for Adverse Events (PRO-CTCAE); Treatment-Induced Neuropathy Assessment Scale (TNAS). N.I. = not investigated or reported. Confirmatory factor analysis (CFA). Exploratory factor analysis (EFA). Root Mean Squared Error of Approximation (RMSEA). K: kappa statistic. * For details about “construct validity” and “responsiveness”, see the original papers referenced in Supplementary Table S2.

**Table 4:** Quality of evidence and recommendations.

PROM				
	Quality of Evidence	Recommendations		Key Strengths/Weaknesses
CAS-CIPN	Low	B	Has the potential to be recommended for use but requires further research	Good internal consistency, but limited data on cross-cultural validity.
CINQ	Very low	C	Not recommended for use	Insufficient reliability and lack of structural validity data.
CIPN Self-check	Very low	C	Not recommended for use	No data on structural validity; limited sample size.
CIPNAT	Moderate	A	Has the potential to be recommended for use but requires further research	Strong internal consistency; moderate structural validity but needs more cultural validation.
CIPNIA-OS	Very low	C	Not recommended for use	Limited evidence on validity; reliability concerns.
CIPN-R-ODS	Moderate	B	Has the potential to be recommended for use but requires further research	Good Rasch analysis results; needs more evidence on responsiveness.
EORTC-QLQ-CIPN20/CIPN15	Moderate	B	Has the potential to be recommended for use but requires further research	Well-studied construct validity; CFA indicates structural validity concerns.
FACT-GOG-Ntx	Moderate	B	Has the potential to be recommended for use but requires further research	Good internal consistency, but CFA showed structural validity issues.
ICPNQ	Low	B	Has the potential to be recommended for use but requires further research	Good reliability for the sensory scale; lower reliability for the autonomic scale.
K-NTX-4	Very low	C	Not recommended for use	Limited construct validity; poor consistency across time points.
L-BASIC	Very low	C	Not recommended for use	Limited sample size; insufficient validity testing.
MOST-S26	Very low	C	Not recommended for use	Lacks evidence on structural validity and responsiveness.
OANQ	Very low	C	Not recommended for use	Insufficient data on all validity measures; small sample size.
PNQ	Very low	C	Not recommended for use	Low internal consistency; needs more evidence on broader applicability.
PRO-CTCAE	Low	B	Has the potential to be recommended for use but requires further research	Good reliability for severity measures; limited evidence on other properties.
TNAS v.1, v.2	Low	B	Has the potential to be recommended for use but requires further research	Acceptable internal consistency; needs more evidence on structural validity.
TNAS v.3	Low	B	Has the potential to be recommended for use but requires further research	High test–retest reliability; limited evidence on construct validity.

Legend: Comprehensive Assessment Scale for Chemotherapy-induced Peripheral Neuropathy (CAS-CIPN); Chemotherapy-Induced Neurotoxicity Questionnaire (CINQ); Chemotherapy-induced peripheral neuropathy self-check sheet (CIPN self-check sheet); Chemotherapy-induced peripheral neuropathy assessment tool (CIPNAT); Chemotherapy-induced peripheral neuropathy integrated assessment—oxaliplatin subscale (CIPNIA-OS); Chemotherapy-induced peripheral neuropathy-Rasch-built Overall Disability Scale (CIPN-R-ODS); European Organization of Research and Treatment of Cancer-Quality of Life Questionnaire-Twenty-item scale (EORTC-QLQ-CIPN15/CIPN20 fifteen/twenty-item scale); Functional Assessment of Cancer Therapy/Gynecologic Oncology Group–Neurotoxicity (FACT-GOG-Ntx); Indication for CTC Grading of Peripheral Neuropathy Questionnaire (ICPNQ); Neurotoxicity 4-item (NTX-4); Location-based assessment of sensory symptoms in cancer (L-BASIC); Measure of Ovarian Symptoms and Treatment-26 items (MOST-S26); Oxaliplatin-Associated Neurotoxicity Questionnaire (OANQ); Patient Neurotoxicity Questionnaire (PNQ); Patient-reported Outcome-Common Terminology Criteria for Adverse Events (PRO-CTCAE); Treatment-Induced Neuropathy Assessment Scale (TNAS). Moderate: Moderately confident in the measurement property estimate; the true measurement property is likely to be close to the estimate of the measurement property, but there is a possibility that it is substantially different. Low: Confidence in the measurement property estimate is limited: the true measurement property may be substantially different from the estimate of the measurement property. Very low: Very little confidence in the measurement property estimate: the true measurement property is likely to be substantially different from the estimate of the measurement property. (A) Instruments with evidence for sufficient content validity (any level) and at least low-quality evidence for sufficient internal consistency. (B) Instruments categorized not in A or C. (C) Instruments with high-quality evidence for an insufficient measurement property.

## Data Availability

The original contributions presented in this study are included in this article and Supplementary Material; further inquiries can be directed to the corresponding author.

## Supplementary Materials

The following supporting information can be downloaded at https://www.mdpi.com/article/10.3390/curroncol31120577/s1: Table S1: Search strategy; Figure S1: PRISMA flow diagram; Table S2: Characteristics of the included studies.
