# Supplementary material for: Peripheral Neuropathy Instruments for Individuals with Cancer: A COSMIN-Based Systematic Review of Measurement Properties
Source: Curr Oncol. 2024 Dec 6;31(12):7828–51. doi: 10.3390/curroncol31120577 (PMC11674663; doi:10.3390/curroncol31120577)
Supplement: Supplementary file 1 [file curroncol-31-00577-s001.zip › Figure S1.pdf]

PRISMA 2020 flow diagram for new systematic reviews which included searches of databases, registers and other sources

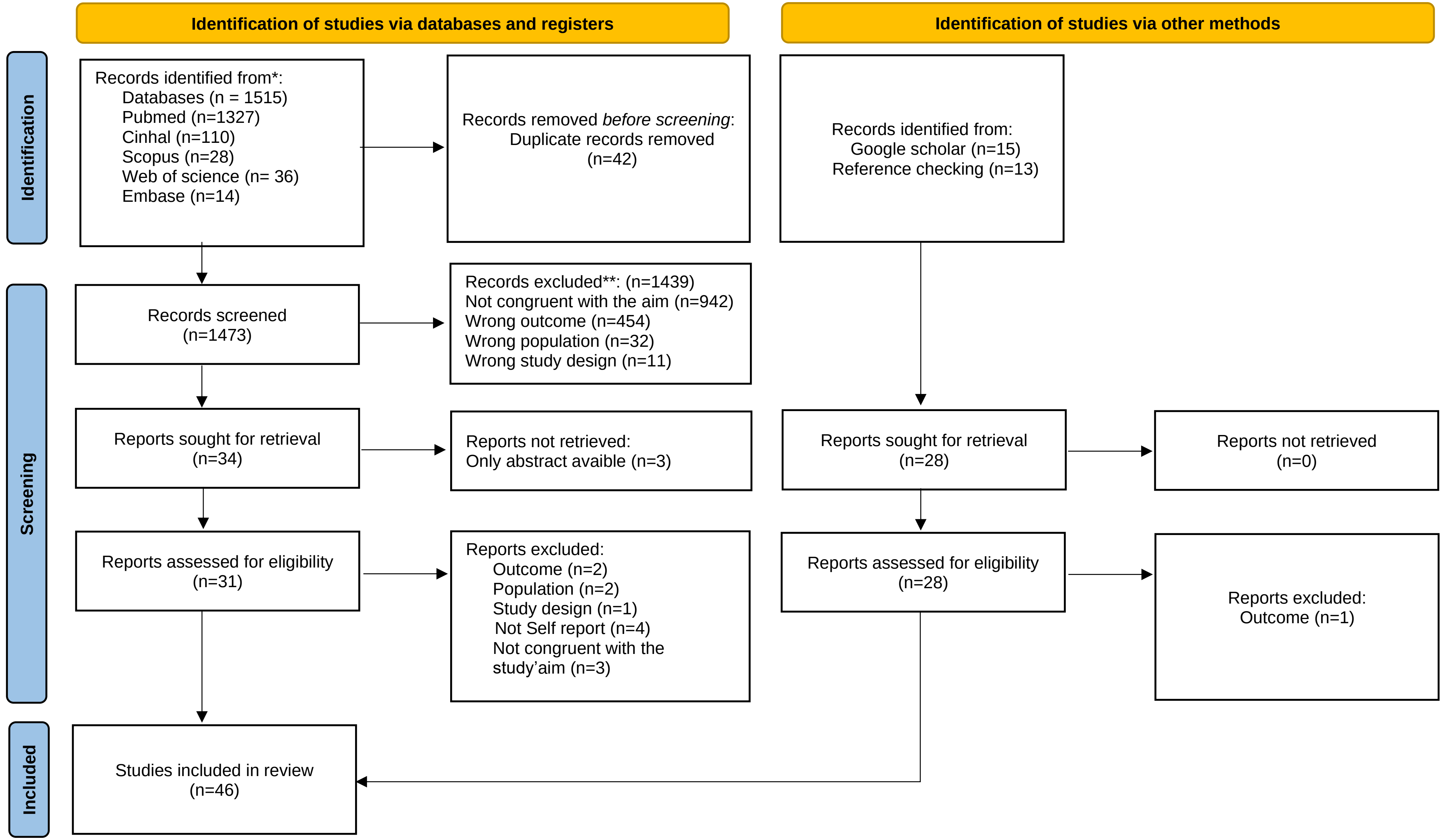

*Consider, if feasible to do so, reporting the number of records identified from each database or register searched (rather than the total number across all databases/registers).
**If automation tools were used, indicate how many records were excluded by a human and how many were excluded by automation tools. *From:* Page MJ, McKenzie JE, Bossuyt PM, Boutron I, Hoffmann TC, Mulrow CD, et al. The PRISMA 2020 statement: an updated guideline for reporting systematic reviews. BMJ 2021;372:n71. doi: 10.1136/bmj.n71.
