# Supplementary material for: Peripheral Neuropathy Instruments for Individuals with Cancer: A COSMIN-Based Systematic Review of Measurement Properties
Source: Curr Oncol. 2024 Dec 6;31(12):7828–51. doi: 10.3390/curroncol31120577 (PMC11674663; doi:10.3390/curroncol31120577)
Supplement: Supplementary file 1 [file curroncol-31-00577-s001.zip › Table S1.pdf]

Table S1. Search strategy.

Database	Query	Date of search	Number of papers
**PubMed**	"Peripheral Nervous System Diseases"[Mesh] OR "Peripheral Nervous System Disease" OR "peripheral neuropathy" OR "PNS Diseases" OR "PNS Disease" OR ‘PNP’ OR ‘CIPN’ OR ‘chemotherapy induced peripheral neuropathy’ OR ‘chemotherapy induced polyneuropathy’ OR ‘polyneuropathy’ OR "Peripheral neuropathies" OR "Peripheral Nerve Diseases" OR "Peripheral Nerve Disease" OR "Peripheral Nervous System Disorders" OR "Chemotherapy-induced peripheral neuropathy" OR "Chemotherapy-Induced Peripheral Neuropathies" OR "Chemotherapy-induced polyneuropathy" OR "CIPN" OR "compression of nerves" OR "compression" OR "nerve damage" OR "nerves damage" OR "postoperative peripheral neuropathy" OR "surgical stress" OR "idiopathic neuropathy" OR compression OR surgery-induced peripheral neuropathy" OR "compression-induced peripheral neuropathy" OR "Compressive Neuropathie*" OR "Nerve Compression Syndrom*" OR "surgery induced peripheral neuropathy" AND "Neoplasms"[Mesh] OR Neoplas* OR Tumor* OR Tumour* OR Cancer* OR Carcino* OR Malignan* OR "Malignant Neoplasms" OR "Malignant Neoplasm" OR oncolog* OR oncog* OR Onkologie OR metasta* OR tumor cells OR tumour cells OR tumor cell OR tumour cell OR cancer cells OR cancer cell OR cell growth OR cancer survival OR cancer control OR advanced cancer OR tumorogen* OR tumorigen* OR tumorgen* AND "Patient Reported Outcome Measures"[Mesh] OR "Patient-Reported Outcome Measure*" OR "Patient Reported Outcome*" OR "Patient-Reported Outcome*" OR "Patient Outcome Assessment"[Mesh] OR "Patient Outcome Assessment*" OR "Patient Outcomes Assessment" OR "Clinical Outcome Assessment*" OR "Clinical Outcomes Assessment*" OR "COA" OR "COAs" OR PROM* OR PREM* OR PRO OR "hr-pro" OR "hr	August 2024	1327

	pro" OR "health index*" OR "health indices" OR instrument* OR questionnaire* OR scale* OR instrument* OR questionnaire* OR scale* OR EORTC QLQ-CIPN20 OR CIPN20 OR scale* OR "comprehensive assessment scale" OR "comprehensive assessment" OR "assessment" OR "measuring outcome*" OR measuring OR "outcome measure*" AND "Psychometrics"[Mesh] OR psychometr* OR "Reproducibility of Results"[Mesh] OR "Reproducibility of Findings" OR "Reproducibility of Result" OR "observer variation"[MeSH] OR "observer variation*" OR "discriminant analysis"[MeSH] OR reliab* OR unreliab* OR valid* OR "coefficient of variation" OR coefficient OR "internal consistency" OR cronbach* OR item* OR correlation* OR agreement* OR alpha* OR agreement* OR reliab* OR interrater* OR "inter-rater" OR intrarater OR "intra-rater" "factor analyses" OR "factor structure" OR "factor structures"OR clinimetric* NOT breastfeeding NOT infant* NOT child* NOT student* NOT caregiver*		
**Cinahl**	TX (peripheral neuropathy or peripheral nerve disease) AND TX (oncology patients or cancer patients or patients with cancer) AND TX (patient reported outcome measures or prom or patient reported outcomes) AND TX (psychometric properties or validity or reliability) NOT TX (children or adolescents or youth or child or teenager) NOT TX (caregivers or family members or relatives or informal caregivers)	August 2024	110
**Scopus**	(TITLE-ABS-KEY ("peripheral neuropathy" OR "peripheral nerve disease") AND TITLE-ABS-KEY (cancer OR neoplasm*) AND TITLE-ABS-KEY ("patient reported outcome" OR prom) AND TITLE-ABS-KEY (psychometrics OR validity OR reliability) AND NOT TITLE-ABS-KEY (child OR young) AND NOT TITLE-ABS-KEY (caregiver*))	August 2024	28
**Web of Science**	peripheral neuropathy or peripheral nerve disease (All Fields) and cancer or neoplasm (All Fields) and patient reported outcome or prom (All Fields) and psychometrics and validity or reliability (All Fields) not pediatric (All Fields) not caregiver (All Fields)	August 2024	36

**Embase**	('peripheral neuropathy'/exp OR 'peripheral neuropathy') AND cancer* AND 'patient-reported outcome' AND 'reliability' AND validity NOT child NOT caregiver	August 2024	14
