# Supplementary material for: Peripheral Neuropathy Instruments for Individuals with Cancer: A COSMIN-Based Systematic Review of Measurement Properties
Source: Curr Oncol. 2024 Dec 6;31(12):7828–51. doi: 10.3390/curroncol31120577 (PMC11674663; doi:10.3390/curroncol31120577)
Supplement: Supplementary file 1 [file curroncol-31-00577-s001.zip › Table S2.pdf]

Supplementary Table 2. General characteristics of the included PROMS

PROM	Country	Population, sample size	Treatment	Construct	Nerves damage	Domains	Longitudinal validity	Items	Intent	Other language
CAS-CIPN [1]	Japan	Various cancers (mostly gastrointestinal), 61 yr, 60,6% female (n=327)	Various chemotherapy (CT) agents (mainly paclitaxel)	CIPN	Sensory, motor, and autonomi c	Physical, emotional, social, spiritual, and behavioural	Not investigated	15	Evaluative	No
CINQ [2]	Netherlands	Various cancers (mostly colon), 62 yr, 56% female (n=143)	Various CT agents (mainly oxaliplatin)	CIPN	Sensory and motor	Physical, social, emotional and functional	Not investigated	n.a	Evaluative	No
CIPN Self-check sheet [3]	Japan	Various cancers (mostly colon), 70 yr, 44% male (n=77)	Various CT agents (mainly oxaliplatin)	CIPN	Sensory and motor	Physical, functional and self-care	Not investigated	14	Evaluative	No
CIPNAT [4–8]	Saudi Arabia	First version: Various cancers, 58 yr ,60% female (n=167)	Various CT agents (mainly paclitaxel)	CIPN	Sensory, motor and autonomi c	Physical and functional	Not investigated	50	Evaluative, discriminative	Turkish, Arabic, Korean
CIPNIA-OS [9]	China	Various cancers, 58 yr, 56% female (n=186)	Various CT agents (mainly oxaliplatin)	CIPN	Sensory and motor	Physical	Investigated	20	Evaluative	No

CIPN-R-ODS [10]	Europe	Various cancers (mostly colon), 64 yr, 62% male (n=281)	Various CT agents (mainly oxaliplatin and paclitaxel)	CIPN	Sensory and motor	Physical, functional and social	Investigated	28	Evaluative	No
EORTC-QLQ-CIPN20/ CIPN15[11–22]	Multi-site worldwide (initial list of items in Dutch	Various cancers, including haematologic malignancies (n=5847)	Various CT agents	CIPN	Sensory, motor and autonomi c	Physical, functional and social	Investigated	20	Evaluative	English French Arabic, Korean
FACT-GOG-Ntx [23–30]	USA	Various cancers, including haematologic malignancies (n=1514)	Various CT agents	CIPN	Sensory, motor, and autonomi c	Physical, functional and social	Investigated	13	Evaluative	Chinese, Chilean
ICPNQ[31]	Europe	Multiple myeloma, 67,5 yr, 71% male (n=156)	Thalidomide, bertezomib, lenalidomid, vincristine and other CT agents	CIPN	Sensory, motor and autonomi c	Physical	Investigated	17	Evaluative	No
K-NTX-4 [32]	Korea	Ovarian, Fallopian tube and primary peritoneal cancer, 54 yr, 100% female (n=250)	Paclitaxel, carboplatin, bleomycin+eto poside and cisplatin (despite exclusion criteria was "chemotherapy within 1 month of study onset"	Neurotoxicity (disease and treatment-related symptoms)	Sensory and motor	Physical, functional, emotional and self-care	Investigated	4	Evaluative	No

L-BASIC [33]	Pennsylvania	Various cancers (mostly breast), 54 yr, 50% female (n=97)	Various CT agents	CIPN, cancer-related head/ neck symptoms, cancer-related breast lymphedema	Sensory	Physical	Investigated	n.a.	Evaluative	No
MOST-S26 [34]	Australia	Ovarian cancer, 60 yr, 100% female (n=790)	Carboplatin and cisplatin	CIPN and ovarian cancer-related symptoms	Not specified (various physical symptoms)	Physical, social, emotional and functional	Investigated	26	Evaluative and surveillance	No
OANQ [35,36]	Sweden	Various cancers (mostly colon), 64 yr, 52% male (n=281)	Oxaliplatin	CIPN	Sensory, motor and autonomi c	Physical and functional	Investigated	29	Evaluative	No
PNQ [37,38]	Japan	Primary validation study [37]: Breast cancer, 52 yr, 100% female (n=300)	Taxanes, cisplatin and carboplatin	CIPN	Sensory and motor	Physical, functional and social	Investigated	2	Evaluative	Greek

PRO-CTCAE-CIPN[39–43]	USA	Both primary validation studies[39,40]: Various cancers (mostly lung, head/neck and gastrointestinal) and myeloma multiple, 57-59 yr, 57%-66% female (n=1082)	Platinum, taxane and proteasome inhibitors, radiotherapy and surgery (mostly CT)	CIPN and radiotherapy-related symptoms	Sensory	Physical, emotional, social, and cognitive	Investigated	2	Evaluative, screening and discriminative	No
TNAS v.1, v.2 [44]	Texas	Multiple myeloma and colorectal cancer, 54-64 yr, 57-62% male (n=573)	Bortezomib and oxaliplatin	CIPN	Sensory and motor	Physical and functional	Investigated	v.1= 11 v.2= 13	Evaluative	No
TNAS v.3[45,46]	Texas	Final version[45]: Various cancers (colorectal, myeloma multiple, gynecologic, 63 yr, 77% women	Bortezomib, oxaliplatin and taxane	CIPN	Sensory and motor	Physical and functional	Not investigated	v.3= 9	Evaluative	No

Comprehensive Assessment Scale for Chemotherapy-induced Peripheral Neuropathy (CAS-CIPN); Chemotherapy Induced Neurotoxicity Questionnaire (CINQ); Chemotherapy-induced peripheral neuropathy self-check sheet (CIPN self-check sheet); Chemotherapy-induced peripheral neuropathy assessment tool (CIPNAT); Chemotherapy-induced peripheral neuropathy integrated assessment – oxaliplatin subscale (CIPNIA-OS); Chemotherapy-induced peripheral neuropathy-Rasch-built Overall Disability Scale (CIPN-R-ODS); European Organization of Research and Treatment of Cancer-Quality of Life Questionnaire-Twenty-item scale (EORTC-QLQ-CIPN15/20 fifteen/twenty-item scale);

Functional Assessment of Cancer Therapy/Gynecologic Oncology Group–Neurotoxicity (FACT/ GOG-Ntx); Indication for CTC Grading of Peripheral Neuropathy Questionnaire (ICPNQ); Neurotoxicity 4-item (NTX-4); Location-based assessment of sensory symptoms in cancer (L-BASIC); Measure of Ovarian Symptoms and Treatment-26 items (MOST-26); Oxaliplatin-Associated Neurotoxicity Questionnaire (OANQ); Patient Neurotoxicity Questionnaire (PNQ); Patient-reported Outcome-Common Terminology Criteria for Adverse Events (PRO-CTCAE); Treatment-Induced Neuropathy Assessment Scale (TNAS).

References

1. Kanda K, Fujimoto K, Mochizuki R, Ishida K, Lee B. Development and validation of the comprehensive assessment scale for chemotherapy-induced peripheral neuropathy in survivors of cancer. *BMC Cancer*. 2019;19(1):904. doi:10.1186/s12885-019-6113-3

2. Driessen CML, de Kleine-Bolt KME, Vingerhoets AJJM, Mols F, Vreugdenhil G. Assessing the impact of chemotherapy-induced peripheral neurotoxicity on the quality of life of cancer patients: the introduction of a new measure. *Support Care Cancer*. 2012;20(4):877-881. doi:10.1007/s00520-011-1336-0

3. Miyoshi Y, Onishi C, Fujie M, Senoo N, Wakatsuki R, Suzumiya J. Validity of the chemotherapy-induced peripheral neuropathy self-check sheet. *Intern Med*. 2015;54(7):737-742. doi:10.2169/internalmedicine.54.3318

4. Jung MS, Kim M, Cha K, Cui X, Lee JW. Psychometric Properties of the Korean Version of the Chemotherapy-Induced Peripheral Neuropathy Assessment Tool. *Res Theory Nurs Pract*. Published online September 12, 2022:RTNP-2022-0037.R1. doi:10.1891/RTNP-2022-0037

5. Kutlutürkan S, Öztürk ES, Arıkan F, Kahraman BB, Özcan K, Uçar MA. The psychometric properties of the Turkish version of the Chemotherapy-Induced Peripheral Neuropathy Assessment Tool (CIPNAT). *Eur J Oncol Nurs*. 2017;31:84-89. doi:10.1016/j.ejon.2017.10.001

6. Obaid A, El-Aqoul A, Alafafsheh A, Abu-Khudair H, Saleh M, Kuliab A. Validation of the Arabic Version of the Chemotherapy-Induced Peripheral Neuropathy Assessment Tool. *Pain Manag Nurs*. 2020;21(6):587-593. doi:10.1016/j.pmn.2020.05.005

7. Simsek NY, Demir A. Reliability and Validity of the Turkish Version of Chemotherapy-induced Peripheral Neuropathy Assessment Tool for Breast Cancer Patients Receiving Taxane Chemotherapy. *Asia Pac J Oncol Nurs*. 2018;5(4):435-441. doi:10.4103/apjon.apjon_29_18

8. Tofthagen CS, McMillan SC, Kip KE. Development and psychometric evaluation of the chemotherapy-induced peripheral neuropathy assessment tool. *Cancer Nurs*. 2011;34(4):E10-20. doi:10.1097/NCC.0b013e31820251de

9. Gu Z, Chen C, Gu J, et al. Development and validation of the chemotherapy-induced peripheral neuropathy integrated assessment – oxaliplatin subscale: a prospective cohort study. *BMC Cancer*. 2023;23(1):1109. doi:10.1186/s12885-023-11541-7

10. Binda D, Vanhoutte EK, Cavaletti G, et al. Rasch-built Overall Disability Scale for patients with chemotherapy-induced peripheral neuropathy (CIPN-R-ODS). *Eur J Cancer*. 2013;49(13):2910-2918. doi:10.1016/j.ejca.2013.04.004

11. Postma TJ, Aaronson NK, Heimans JJ, et al. The development of an EORTC quality of life questionnaire to assess chemotherapy-induced peripheral neuropathy: the QLQ-CIPN20. *Eur J Cancer*. 2005;41(8):1135-1139. doi:10.1016/j.ejca.2005.02.012

12. Lavoie Smith EM, Barton DL, Qin R, Steen PD, Aaronson NK, Loprinzi CL. Assessing patient-reported peripheral neuropathy: the reliability and validity of the European Organization for Research and Treatment of Cancer QLQ-CIPN20 Questionnaire. *Qual Life Res*. 2013;22(10):2787-2799. doi:10.1007/s11136-013-0379-8

13. Liu H, Tan AD, Qin R, et al. Comparing and Validating Simple Measures of Patient-Reported Peripheral Neuropathy for Oncology Clinical Trials: NCCTG N0897 (Alliance) A Pooled Analysis of 2440 Patients. *SOJ Anesthesiol Pain Manag*. 2015;2(2):10.15226/2374-684X/2/2/00120.

14. Kieffer JM, Postma TJ, van de Poll-Franse L, et al. Evaluation of the psychometric properties of the EORTC chemotherapy-induced peripheral neuropathy questionnaire (QLQ-CIPN20). *Qual Life Res*. 2017;26(11):2999-3010. doi:10.1007/s11136-017-1626-1

15. Lavoie Smith EM, Haupt R, Kelly JP, et al. The Content Validity of a Chemotherapy-Induced Peripheral Neuropathy Patient-Reported Outcome Measure. *Oncol Nurs Forum*. 2017;44(5):580-588. doi:10.1188/17.ONF.580-588

16. Smith EML, Knoerl R, Yang JJ, Kanzawa-Lee G, Lee D, Bridges CM. In Search of a Gold Standard Patient-Reported Outcome Measure for Use in Chemotherapy- Induced Peripheral Neuropathy Clinical Trials. *Cancer Control*. 2018;25(1):1073274818756608. doi:10.1177/1073274818756608

17. Lavoie Smith EM, Zanville N, Kanzawa-Lee G, et al. Rasch model-based testing of the European Organisation for Research and Treatment of Cancer (EORTC) Quality of Life Questionnaire-Chemotherapy-Induced Peripheral Neuropathy (QLQ-CIPN20) using Alliance for Clinical Trials in Oncology (Alliance) A151408 study data. *Support Care Cancer*. 2019;27(7):2599-2608. doi:10.1007/s00520-018-4553-y

18. Lavoie Smith EM, Banerjee T, Yang JJ, et al. Psychometric Testing of the European Organisation for Research and Treatment of Cancer (EORTC) QLQ-CIPN20 Using Pooled Chemotherapy-Induced Peripheral Neuropathy Outcome Measures Standardization (CI-PeriNomS) and Alliance for Clinical Trials in Oncology (Alliance) A151408 Study Data. *Cancer Nurs*. 2019;42(3):179-189. doi:10.1097/NCC.0000000000000596

19. Cavaletti G, Cornblath DR, Merkies ISJ, et al. The chemotherapy-induced peripheral neuropathy outcome measures standardization study: from consensus to the first validity and reliability findings. *Ann Oncol*. 2013;24(2):454-462. doi:10.1093/annonc/mds329

20. Abu Sharour L. Psychometric evaluation of the Arabic Version the European Organization for Research and Treatment of Cancer Quality of Life Questionnaire for Chemotherapy-Induced Peripheral Neuropathy Questionnaire (EORTC QLQ-CIPN20). *Appl Neuropsychol Adult*. 2021;28(5):614-618. doi:10.1080/23279095.2019.1677232

21. Kim HY, Kang JH, Youn HJ, et al. Reliability and validity of the Korean version of the European Organization for Research and Treatment of Cancer Quality of Life Questionnaire to assess Chemotherapy-induced peripheral neuropathy. *J Korean Acad Nurs*. 2014;44(6):735-742. doi:10.4040/jkan.2014.44.6.735

22. Yeo F, Ng CC, Loh KWJ, et al. Minimal clinically important difference of the EORTC QLQ-CIPN20 for worsening peripheral neuropathy in patients receiving neurotoxic chemotherapy. *Support Care Cancer*. 2019;27(12):4753-4762. doi:10.1007/s00520-019-04771-8

23. Calhoun EA, Welshman EE, Chang CH, et al. Psychometric evaluation of the Functional Assessment of Cancer Therapy/Gynecologic Oncology Group-Neurotoxicity (Fact/GOG-Ntx) questionnaire for patients receiving systemic chemotherapy. *Int J Gynecol Cancer*. 2003;13(6):741-748. doi:10.1111/j.1525-1438.2003.13603.x

24. Cheng HL, Lopez V, Lam SC, et al. Psychometric testing of the Functional Assessment of Cancer Therapy/Gynecologic Oncology Group-Neurotoxicity (FACT/GOG-Ntx) subscale in a longitudinal study of cancer patients treated with chemotherapy. *Health Qual Life Outcomes*. 2020;18(1):246. doi:10.1186/s12955-020-01493-y

25. Kaiser K, Lyleroehr M, Shaunfield S, et al. Neuropathy experienced by colorectal cancer patients receiving oxaliplatin: A qualitative study to validate the Functional Assessment of Cancer Therapy/Gynecologic Oncology Group-Neurotoxicity scale. *World J Gastrointest Oncol*. 2020;12(2):205-218. doi:10.4251/wjgo.v12.i2.205

26. Ribeiro IL, Lorca LA, Cuevas-Cid R, Dixit S, Yáñez-Benavides N, Ortega-Gonzalez F. Validation of the Functional Assessment of Cancer Therapy/Gynecologic Oncology Group Neurotoxicity Questionnaire for the Latin American Population. *Int J Breast Cancer*. 2022;2022:6533797. doi:10.1155/2022/6533797

27. Cheng HL, Molassiotis A. Longitudinal validation and comparison of the Chinese version of the European Organization for Research and Treatment of Cancer Quality of Life-Chemotherapy-Induced Peripheral Neuropathy Questionnaire (EORTC QLQ-CIPN20) and the Functional Assessment of Cancer-Gynecologic Oncology Group-Neurotoxicity subscale (FACT/GOG-Ntx). *Asia Pac J Clin Oncol*. 2019;15(1):56-62. doi:10.1111/ajco.13000

28. Huang HQ, Brady MF, Cella D, Fleming G. Validation and reduction of FACT/GOG-Ntx subscale for platinum/paclitaxel-induced neurologic symptoms: a gynecologic oncology group study. *Int J Gynecol Cancer*. 2007;17(2):387-393. doi:10.1111/j.1525-1438.2007.00794.x

29. Cella D, Peterman A, Hudgens S, Webster K, Socinski MA. Measuring the side effects of taxane therapy in oncology: the functional assesment of cancer therapy-taxane (FACT-taxane). *Cancer*. 2003;98(4):822-831. doi:10.1002/cncr.11578

30. Alberti P, Bernasconi DP, Cornblath DR, et al. Prospective Evaluation of Health Care Provider and Patient Assessments in Chemotherapy-Induced Peripheral Neurotoxicity. *Neurology*. 2021;97(7):e660-e672. doi:10.1212/WNL.0000000000012300

31. Beijers AJM, Vreugdenhil G, Oerlemans S, et al. Chemotherapy-induced neuropathy in multiple myeloma: influence on quality of life and development of a questionnaire to compose common toxicity criteria grading for use in daily clinical practice. *Support Care Cancer*. 2016;24(6):2411-2420. doi:10.1007/s00520-015-3032-y

32. Lee M, Lee Y, Kim K, et al. Development and Validation of Ovarian Symptom Index-18 and Neurotoxicity-4 for Korean Patients with Ovarian, Fallopian Tube, or Primary Peritoneal Cancer. *Cancer Res Treat*. 2019;51(1):112-118. doi:10.4143/crt.2017.361

33. Burkey AR, Kanetsky PA. Development of a novel location-based assessment of sensory symptoms in cancer patients: preliminary reliability and validity assessment. *J Pain Symptom Manage*. 2009;37(5):848-862. doi:10.1016/j.jpainsymman.2008.05.013

34. Campbell R, King MT, Ross TL, Cohen PA, Friedlander ML, Webb PM. Development and validation of the measure of ovarian symptoms and treatment concerns for surveillance (MOST-S26): An instrument to complement the clinical follow-up of women with ovarian cancer after completion of first-line treatment. *Gynecol Oncol*. 2021;163(2):398-407. doi:10.1016/j.ygyno.2021.08.022

35. Gustafsson E, Litström E, Berterö C, Drott J. Reliability testing of oxaliplatin-associated neurotoxicity questionnaire (OANQ), a pilot study. *Support Care Cancer*. 2016;24(2):747-754. doi:10.1007/s00520-015-2838-y

36. Leonard GD, Wright MA, Quinn MG, et al. Survey of oxaliplatin-associated neurotoxicity using an interview-based questionnaire in patients with metastatic colorectal cancer. *BMC Cancer*. 2005;5:116. doi:10.1186/1471-2407-5-116

37. Shimozuma K, Ohashi Y, Takeuchi A, et al. Feasibility and validity of the Patient Neurotoxicity Questionnaire during taxane chemotherapy in a phase III randomized trial in patients with breast cancer: N-SAS BC 02. *Support Care Cancer*. 2009;17(12):1483-1491. doi:10.1007/s00520-009-0613-7

38. Tsoleridis T, Chloropoulou P, Tsaroucha A, Vadalouca A, Siafaka I, Vogiatzaki T. Validation of the Patient Neurotoxicity Questionnaire for Patients Suffering From Chemotherapy-Induced Peripheral Neuropathy in Greek. *Cureus*. 2021;13(4):e14324. doi:10.7759/cureus.14324

39. Knoerl R, Mazzola E, Mitchell SA, et al. Measurement properties of brief neuropathy screening items in cancer patients receiving taxanes, platinums, or proteasome inhibitors. *J Patient Rep Outcomes*. 2021;5(1):101. doi:10.1186/s41687-021-00377-z

40. Dueck AC, Mendoza TR, Mitchell SA, et al. Validity and Reliability of the US National Cancer Institute's Patient-Reported Outcomes Version of the Common Terminology Criteria for Adverse Events (PRO-CTCAE). *JAMA Oncol*. 2015;1(8):1051-1059. doi:10.1001/jamaoncol.2015.2639

41. Hay JL, Atkinson TM, Reeve BB, et al. Cognitive interviewing of the US National Cancer Institute's Patient-Reported Outcomes version of the Common Terminology Criteria for Adverse Events (PRO-CTCAE). *Qual Life Res*. 2014;23(1):257-269. doi:10.1007/s11136-013-0470-1

42. Knoerl R, Gray E, Stricker C, et al. Electronic versus paper-pencil methods for assessing chemotherapy-induced peripheral neuropathy. *Support Care Cancer*. 2017;25(11):3437-3446. doi:10.1007/s00520-017-3764-y

43. Basch E, Reeve BB, Mitchell SA, et al. Development of the National Cancer Institute's patient-reported outcomes version of the common terminology criteria for adverse events (PRO-CTCAE). *J Natl Cancer Inst*. 2014;106(9):dju244. doi:10.1093/jnci/dju244

44. Mendoza TR, Wang XS, Williams LA, et al. Measuring Therapy-Induced Peripheral Neuropathy: Preliminary Development and Validation of the Treatment-Induced Neuropathy Assessment Scale. *J Pain*. 2015;16(10):1032-1043. doi:10.1016/j.jpain.2015.07.002

45. Mendoza TR, Williams LA, Shi Q, et al. The Treatment-induced Neuropathy Assessment Scale (TNAS): a psychometric update following qualitative enrichment. *J Patient Rep Outcomes*. 2020;4(1):15. doi:10.1186/s41687-020-0180-8

46. Williams LA, Garcia-Gonzalez A, Mendoza TR, Haq S, Cleeland CS. Concept domain validation and item generation for the Treatment-Induced Neuropathy Assessment Scale (TNAS). *Support Care Cancer*. 2019;27(3):1021-1028. doi:10.1007/s00520-018-4391-y
